# Synthesis of Imidazole-Compound-Coated Copper Nanoparticles with Promising Antioxidant and Sintering Properties

**DOI:** 10.3390/mi14112079

**Published:** 2023-11-09

**Authors:** Yu Zhang, Xianchong Yu, Ziyuan Chen, Song Wu, Haiqi Lai, Shiwo Ta, Tingyu Lin, Guannan Yang, Chengqiang Cui

**Affiliations:** 1 State Key Laboratory of Precision Electronic Manufacturing Technology and Equipment, Guangdong University of Technology, Guangzhou 510006, China; zhangyu@gdut.edu.cn (Y.Z.); 2112101387@mail2.gdut.edu.cn (X.Y.); ziyuan.chen@hatchip.com (Z.C.); 18296309432@163.com (S.W.); hykih345@gmail.com (H.L.); 2 State Key Laboratory of Advanced Materials and Electronic Components, Fenghua Advanced Technology Co., Ltd., Zhaoqing 526020, China; tashiwo@china-fenghua.com; 3 Guangdong Fozhixin Microelectronics Technology Research Co., Ltd., Foshan 528200, China; tingyulin@fzxsmc.com

**Keywords:** Cu nanoparticles, imidazole compounds, sintering properties, oxidation resistance, complexing effect

## Abstract

In this study, we present a facile method for preparing oxidation-resistant Cu nanoparticles through a liquid-phase reduction with imidazole compounds (imidazole, 2-methylimidazole, 2-phenylimidazole, and benzimidazole) that serve as protective and dispersing agents. Through a complexation reaction between Cu atoms, the imidazole compounds can form a protective film on the Cu nanoparticles to prevent the particles from rapidly oxidizing. We compared the effects of the four kinds of imidazole compounds on the oxidation resistance and sintering properties of Cu particles. The Cu particles prepared with benzimidazole could be stored in the air for 30 days without being oxidized. After sintering at 300 °C and 2 MPa, the joint of the particles could reach a shear strength of 32 MPa, which meets the requirements for microelectronic packaging.

## 1. Introduction

With excellent electrical and sintering properties, Cu nanoparticles are regarded as a promising material in the fields of microelectronic packaging and interconnects [1,2]. Due to the high surface energy and size effect of nanoparticles [3,4], Cu nanoparticles can be sintered at low temperatures and operated at high temperatures. These characteristics render them potential interconnect materials for power electronics in extreme working conditions [5,6]. However, Cu nanoparticles are easily oxidized in the air [7]. Cupric oxides have a higher melting point and resistivity than pure Cu, which hinders the formation of sintering necks between Cu particles, resulting in poor sintering properties and low conductivity [8,9,10]. The method of fabricating Cu nanoparticles with strong oxidation resistance has become a crucial question for their application in electronics manufacturing [11,12,13].

At present, various coating methods have been proposed to improve the oxidation resistance of Cu nanoparticles [14]. The coating methods can be divided into organic and inorganic coatings. The materials for organic coating treatments use organic protective agents to form a thin film on the particles during the synthetic process [15]. For example, Thi et al. used PVP as the protective agent, cupric sulfate as the precursor, ascorbic acid as the antioxidant, and sodium borohydride as the reducing agent to synthesize Cu nanoparticles with a diameter of 10 nm [16]. The as-prepared Cu nanoparticle colloid remained stable in an organic solution for 2 months. Li et al. used PVP as the protective agent, copper sulfate pentahydrate as the precursor, and hydrazine hydrate as the reducing agent to prepare Cu nanoparticles with a diameter of 200 nm in an aqueous solution [17]. The as-prepared Cu nanoparticles could be stored stably for 2 weeks at room temperature. Wei et al. used lauric acid as the protective agent, copper chloride as the precursor, and NaBH4 as the reducing agent to prepare Cu nanoparticles with a diameter of 3.3 ± 0.7 nm in an aqueous solution [18]. The as-prepared Cu nanoparticles could be stored stably for 2 days at room temperature.

Inorganic coating treatments use inorganic elements, such as silver [19,20], phosphorus [21], and tin [13], to form a compact protective film on the Cu particles. For example, in a study by Dai et al., cupric formate was heated to 170 °C in an oleylamine solution and was decomposed to form Cu nanoparticles [22]. Then, silver nitrate was added to the solution and reacted with the Cu nanoparticles to form Ag-coated Cu nanoparticles. The as-prepared particles could be stored in the air for 2 weeks without being oxidized. In a study by Yang et al., Ag-coated Cu nanoparticles were obtained by adding Cu nanoparticles into a solution with silver nitrate, ammonia, and a complexing agent [19,20]. The obtained particles were almost free of oxidation after exposure to the air for 48 days.

However, the above methods also have their disadvantages. The preservation time of organic-coated Cu nanoparticles in the air is still limited (usually less than 2 weeks), and the residues of many organic agents are difficult to remove and are not conducive to sintering [23]. The inorganic coating method faces the problems of a loose and uneven coating, a complicated process, and a high cost. For these reasons, new methods are required to provide Cu nanoparticles with good oxidation resistance while not limiting their sintering ability [24].

In the literature or industrial requirements, the sintering temperature and pressure for Cu nanoparticles usually fall in the range of 250~300 °C and 1~20 MPa, respectively [25]. Some studies also used sintering conditions similar to those in this paper. For example, Dai et al. [26] reported a shear strength of 30 MPa for Cu-Cu joints under the sintering conditions of 300 °C and 5 MPa pressure. Chen et al. [27] reported a robust bonding strength of 56.7 MPa for SiC chips on Cu pads after sintering at 300 °C and 2 MPa. Susumu Arai et al. [28] reported a shear strength of 34 MPa for Cu-Cu joints after sintering at 300 °C and 10 MPa. However, the performance of the Cu nanoparticles after long-term storage was not provided, which is an important issue for their industrial applications.

It is notable that organic solderability preservatives (OSPs) are widely used in the antioxidation treatment of printed circuit boards (PCBs). Imidazole compounds are the main film-forming materials in OSPs. Through complexation reactions with metallic Cu, the imidazole compounds can form a compact organic coordination polymer film on the bare copper surface, which can protect the bare copper surface from being oxidized or corroded [29,30,31,32,33]. Although the OSP treatment has been widely used and proven to be effective on the copper circuits of PCBs, the effect of an imidazole compound coating on copper nanoparticles is rarely explored. Therefore, in this study, we developed a facile synthetic method for fabricating imidazole-compound-coated Cu nanoparticles. As a small molecular organic compound, imidazoles can be decomposed at a relatively low temperature and can react with rosin in the sintering flux of Cu paste without producing organic residues. When using this method, the imidazole-coated Cu nanoparticles possessed good oxidation resistance and sintering properties, with a size below 500 nm.

## 2. Materials and Methods

PVP K30 (average molecular weight = 40,000), copper acetate (C_4_H_6_CuO_4_·H_2_O), ascorbic acid, anhydrous ethanol, imidazole, 2-methylimidazole, 2-phenylimidazole, and benzimidazole were purchased from Aladdin Biochemical Technology Co., LTD, Shanghai, China. Terpineol (C_10_H_18_O), colophony (C_19_H_29_COOH), and cetyltrimethyl ammonium bromide (C_19_H_42_BrN, CTAB) were purchased from Macklin Inc., Shanghai, China. All chemicals were used as received without further purification.

To form the coated Cu nanoparticles, first, 0.05 mol of copper acetate and 0.00625 mol of protective/dispersing agent (imidazole, 2-methylimidazole, 2-phenylimidazole, benzimidazole, or PVP) were dissolved in 100 mL of anhydrous ethanol. Then, 0.2 mol of ascorbic acid was dissolved in another 150 mL of anhydrous ethanol. The two solutions were mixed and mechanically stirred at 60 °C for 15 min. As a reducing agent, ascorbic acid reacts with copper acetate to reduce copper ions into metallic copper (${Cu({CH}_{3}HCOO)}_{2}\cdot H_{2}O+C_{6}H_{8}O_{6}\to Cu+C_{6}H_{6}O_{6}+{CH}_{3}COOH↑$), thereby forming copper particles. The Cu particles were extracted from the solution via centrifugation at 12,000 rpm for 3 min and then dried in a vacuum for 3 h.

Prior to their use, Cu substrates (10 × 10 × 0.8 mm^3^) and dummy chips (4 × 4 × 0.8 mm^3^) were washed with 0.05 M sulfuric acid and deionized water, and then dried. The Cu paste was prepared by mixing Cu nanoparticles, terpineol, colophony, and CTAB at a weight ratio of 80:16.5:3:0.5. Then, the Cu paste was coated onto the Cu substrates using a spreader with a gap height of 20 μm, and covered using the dummy chips. The samples were then sintered at 300 °C and 2 MPa for 30 min in an Ar–H2 (95:5) atmosphere with a thermocompressor (VHP-5000N-2, KJ Group, Shanghai, China). After sintering, an IC Package Soldering Strength Tester (SERIES-4000-DONDESTER, Dage, London, UK) was used to test the shear strength of the samples under a shear rate of 0.1 mm/min. The shear strength for each sample was obtained by dividing the measured shear force by the area of the joint layer. At least five samples were tested for each data point.

The resistivities of the sintered Cu nanoparticles with and without coating agents were measured via a standard four-point probe method using a four-point probe system (Guangzhou Four Probe Technology Co., Ltd., ST-102E, Guangzhou, China) with a source meter unit (Keithley, 6220, Cleveland, OH, USA). The Cu nanoparticle pastes were coated onto silicon substrates and covered with silicon plates. The thickness of the nanoparticle pastes was controlled at ~20 μm. Then, the samples were sintered under the same conditions of 300 °C and 2 MPa for 30 min in an Ar–H_2_ (95:5) atmosphere. Then, the upper silicon layer was removed. Two constant current probes were fixed at two points of the sintered layer, with a distance of 9 mm. Two voltage probes were fixed at the trisection points between the current probes. The resistivity (*ρ*) could then be obtained based on the voltage (*V*), current (*I*), and thickness of the sintered layer (*W*) according to the equation:

(1) $$\rho =\frac{\pi W}{ln2}\cdot \frac{V}{I}$$

Field emission scanning electron microscopy (FE-SEM; SU8220, Hitachi, Tokyo, Japan) was used to characterize the morphology of the Cu nanoparticles and the failure surface of the Cu–Cu interconnect joints. X-ray diffractometry (XRD; Bruker D8 ADVANCE, Karlsruhe, Germany) was used to observe the phase composition of the Cu nanoparticles. A thermogravimetric analyzer (TGA; Mettler, Toledo, Zurich, Switzerland) was used to analyze the weight change of the Cu nanoparticle pastes over 25–600 °C in a N_2_ atmosphere and in an air atmosphere with a heating rate of 10 °C·min^−1^. Fourier-transform infrared spectroscopy (FTIR; Nicolet IS50, Thermofisher, Waltham, MA, USA) was used to observe the presence of organic groups on the surface of the Cu nanoparticles.

## 3. Results and Discussion

**Figure 1:**
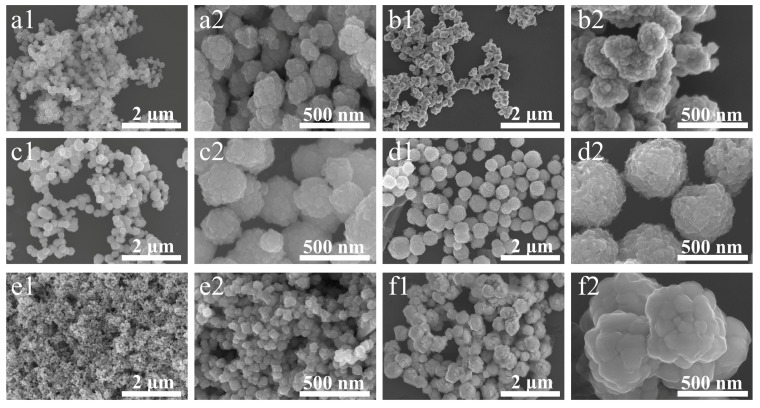
FE-SEM images of the morphologies of the synthesized Cu nanoparticles with (**a1**,**a2**) imidazole, (**b1**,**b2**) 2-methylimidazole, (**c1**,**c2**) 2-phenylimidazole, (**d1**,**d2**) benzimidazole, (**e1**,**e2**) PVP, and (**f1**,**f2**) no protection agent.

Figure 1 shows SEM images of the morphologies of the synthesized Cu nanoparticles with imidazole, 2-methylimidazole, 2-phenylimidazole, and benzimidazole PVP, or without protective agents. The Cu particles are spherical or in ellipsoid shapes and are dispersed. The particle sizes of the six kinds of Cu nanoparticles are ~200, ~250, ~300, ~500, ~100, and ~600 nm, respectively.

**Figure 2:**
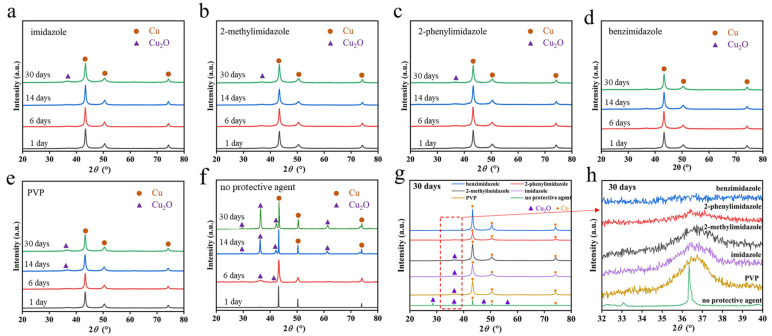
XRD patterns of the synthesized Cu nanoparticles with (**a**) imidazole, (**b**) 2-methylimidazole, (**c**) 2-phenylimidazole, (**d**) benzimidazole, (**e**) PVP, and (**f**) no protective agent after exposure in air for 1, 6, 14, and 30 days. (**g**) Comparison between the XRD patterns of the 6 samples after exposure in air for 30 days. (**h**) Enlarged XRD patterns near 2θ = 36.5°.

Figure 2 shows the XRD patterns of the synthesized Cu nanoparticles with imidazole, 2-methylimidazole, 2-phenylimidazole, benzimidazole, and PVP, or without protective agents, after exposure in the air for 1, 6, 14, and 30 days. Three diffraction peaks can be observed on all the XRD patterns at 2*θ* = 43.38, 50.43, and 74.15°, corresponding to the (111), (200), and (220) crystal planes of metallic Cu, respectively. For the sample without a protective agent, obvious peaks of cuprous oxide can be observed at 2*θ* = 29.45°, 36.502°, 42.23°, and 61.20°, indicating the poor oxidation resistance of the Cu nanoparticles. For the samples with imidazole, 2-methylimidazole, and 2-phenylimidazole, a small peak of cuprous oxide can be noted at 2*θ* = 36.502° after 30 days of exposure. The sample with PVP shows the cuprous oxide peak earlier after 6 days of exposure. The Cu nanoparticles with the benzimidazole protective agent do not show the peaks of oxides after 30 days of exposure, confirming the good oxidation resistance provided by the benzimidazole protective agent.

**Figure 3:**
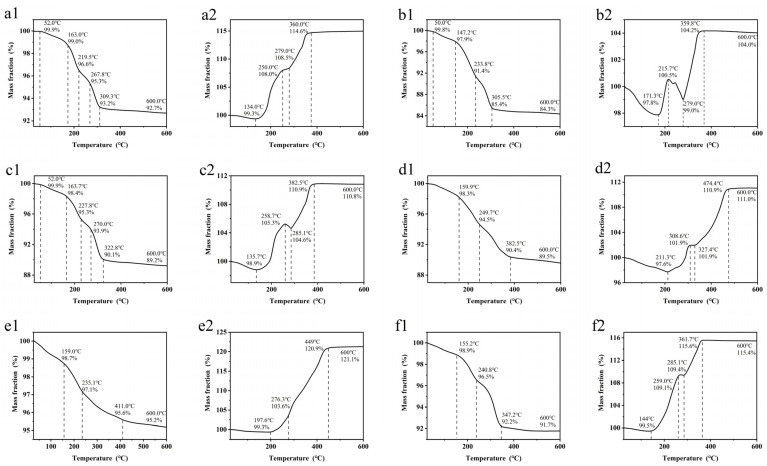
TGA curves of the Cu nanoparticles with (**a1**) imidazole in N_2_, (**a2**) imidazole in air, (**b1**) 2-methylimidazole in N_2_, (**b2**) 2-methylimidazole in air, (**c1**) 2-phenylimidazole in N_2_, (**c2**) 2-phenylimidazole in air, (**d1**) benzimidazole in N_2_, (**d2**) benzimidazole in air, (**e1**) PVP in N_2_, (**e2**) PVP in air, (**f1**) no protective agent in N_2_, and (**f2**) no protective agent in air.

Figure 3 shows the TGA curves of the Cu nanoparticles with the five protective agents and no protective agent, measured in N_2_ and in the air. For all the samples measured in N_2_, the curves drop relatively steeply before reaching a temperature of 300–400 °C, corresponding to the decomposition of the residual organic materials. After the organic decomposition, the weight loss slows. According to the final weight fraction, the Cu content in the Cu nanoparticles is estimated to be 84–95%. The TGA curves of the samples in the air start to increase at 130–210 °C, corresponding to the oxidation of Cu. The curves of 2-methylimidazole (Figure 3(b2)) and 2-phenylimidazole (Figure 3(c2)) show a small drop at 215.7–279 °C and 258.7–285.1 °C, respectively. These results indicate that the decomposition of the coating agents is faster than the oxidation of Cu over this temperature range. Above 360–470 °C, the curves reach a plateau, corresponding to the end of the oxidation process. In comparison, the decomposition temperature in N_2_ and the oxidation temperature of the Cu nanoparticles with the benzimidazole protective agent are relatively higher (Figure 3(d1,d2)), indicating that the Cu nanoparticles with this protective agent have better oxidation resistance. The mass reduction of the Cu nanoparticles without a protective agent in N_2_ is probably due to the use of excessive ascorbic acid and other organic impurities in the solvent during the preparation process, as some organic residues might remain on the surface of the copper particles, causing the mass loss in the subsequent thermogravimetric experiments.

**Figure 4:**
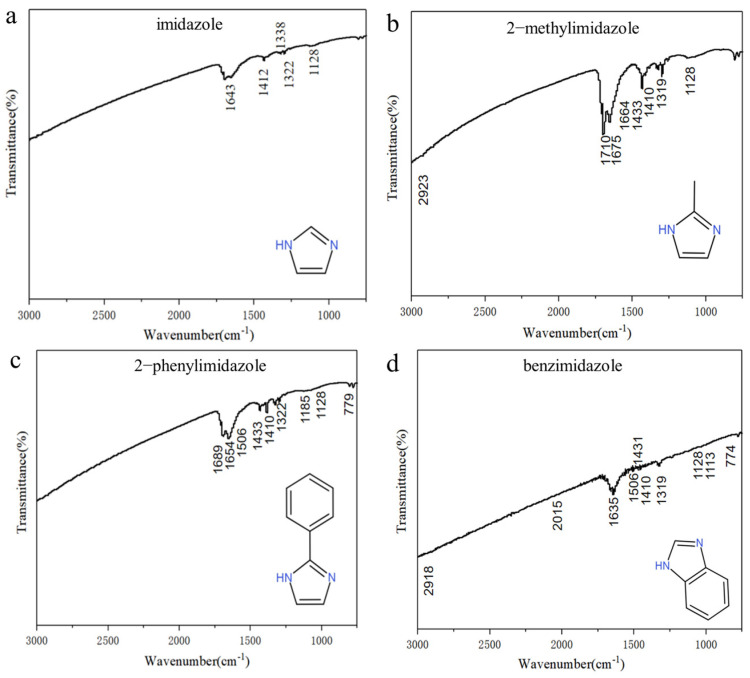
FTIR spectra of the prepared Cu nanoparticles with (**a**) imidazole, (**b**) 2-methylimidazole, (**c**) 2-phenylimidazole, and (**d**) benzimidazole protective agents.

Figure 4 shows the FTIR spectra of the prepared Cu nanoparticles with each of the four imidazole-based compounds. For the Cu nanoparticles with imidazole (Figure 4a), the peak at 1643 cm^−1^ represents the stretching vibration of (–C=C–) and (–C=N–). The peak at 1412 cm^−1^ represents the vibration of the C–H bond in (–CH_2_–). The peaks at 1338, 1322, and 1128 cm^−1^ represent the vibration of (–CN–), confirming the existence of imidazole. For the Cu nanoparticles with 2-methylimidazole (Figure 4b), the peaks at 1710, 1675, and 1664 cm^−1^ represent the stretching vibrations of (–C=C–) and (–C=N–). The peaks at 1433 and 1410 cm^−1^ represent the deformation vibration of the CH bond in (–CH_2_–). The peaks at 1319 and 1128 cm^−1^ represent the vibration of (–CN–). The curve confirms the existence of 2-methylimidazole. For the Cu nanoparticles with 2-phenylimidazole (Figure 4c), the peaks at 1689 and 1654 cm^−1^ represent the stretching vibrations of (–C=C–) and (–C=N–). The peaks at 1433 and 1410 cm^−1^ represent the deformation vibration of the CH bond in (–CH_2_–). The peak at 1322 cm^−1^ represents the vibration of (–CN–). The peak at 779 cm^−1^ represents the out-of-plane bending vibration of the hydrogens in a benzene ring. The spectrum confirms the existence of 2-phenylimidazole. For the Cu nanoparticles with benzimidazole (Figure 4d), the peaks at 1635, 1319, and 774 cm^−1^ correspond to the stretching vibrations of (–C=C–) and of (–CN–) and the out-of-plane bending vibrations of the hydrogens in a benzene ring, respectively. The spectrum shows the presence of benzene rings and confirms that the Cu nanoparticles contain benzimidazole-related substances.

The resistivities of the sintered Cu nanoparticles were measured and are summarized in Table 1. The sintered Cu nanoparticles without a protective agent show a resistivity of 30.15 μΩ·cm, which is probably caused by oxidation during the sample preparation process. The sintered Cu nanoparticles with benzimidazole show the lowest resistivity of 4.86 μΩ·cm among the samples, which is consistent with its best oxidation resistivity. With good electrical conductivity, the benzimidazole treatment could be considered a feasible approach for the application of Cu nanoparticles in microelectronic packaging.

**Figure 5:**
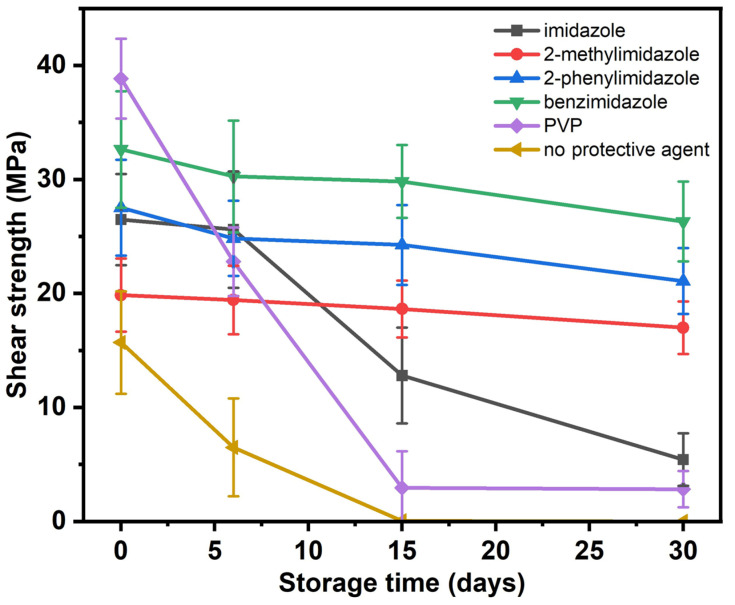
Shear strengths of the sintered Cu nanoparticle joints after storage for 0, 6, 15, and 30 days.

The Cu nanoparticles with the five protective agents and that with no protective agent were stored in the air for 0–30 days and then sintered at 300 °C and 2 MPa for 30 min. To determine the shear strengths of the sintered Cu nanoparticle joints, 10 samples were measured for each sintering condition, and the average values of the results were taken and are summarized in Figure 5. The errors were derived based on the standard deviation of the results. It was found that the strength of all the samples decreased with storage time due to the oxidation of the particles. The strength of the samples with PVP and without a protective agent decreased more sharply with storage time than the others due to their poor oxidation resistance. In comparison, the samples with benzimidazole maintained a relatively high strength after 30 days of storage, which was consistent with their better oxidation resistance, as shown in the XRD patterns in Figure 2.

**Figure 6:**
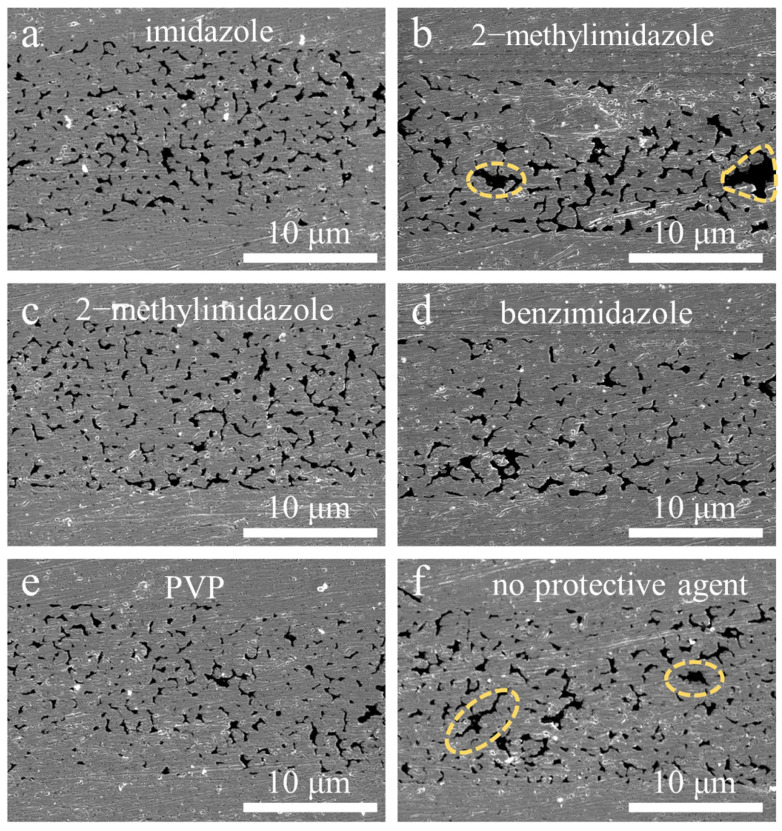
Cross-section images of the sintered Cu nanoparticle joints with the protective agents of (**a**) imidazole, (**b**) 2-methylimidazole, (**c**) 2-phenylimidazole, (**d**) benzimidazole, and (**e**) PVP, or (**f**) without protective agent. The dashed circles indicate the locations of large-scale voids.

**Figure 7:**
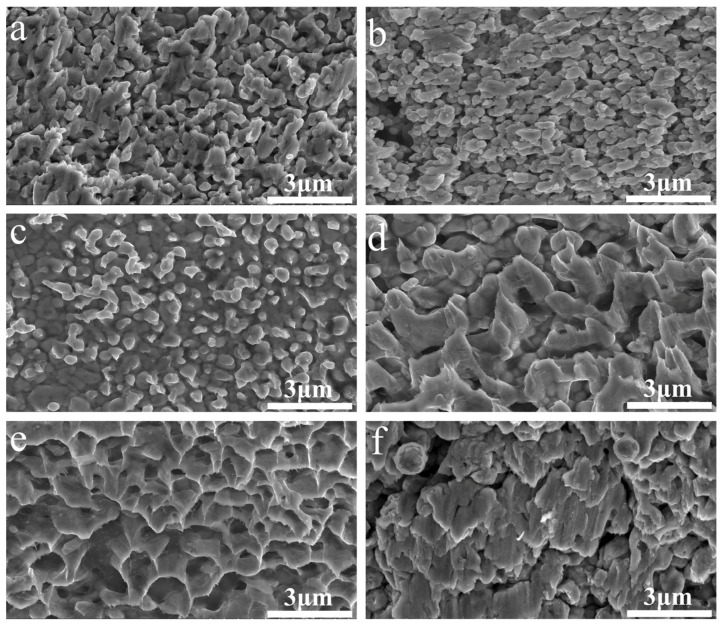
Fracture surfaces of the sintered joints with the protective agents (**a**) imidazole, (**b**) 2-methylimidazole, (**c**) 2-phenylimidazole, (**d**) benzimidazole, and (**e**) PVP, or (**f**) without a protective agent.

Figure 6 shows FE-SEM images of the cross-sections of the joints of the sintered Cu nanoparticles produced with different protective agents and without a protective agent prior to storage. The Cu nanoparticles formed sintering necks with adjacent particles and merged with the upper and lower substrates. For the samples produced with 2-methylimidazole and without a protective agent, large-scale voids could be observed in the cross-section, which was consistent with the relatively low shear strength of the samples. The fracture surfaces of the samples are also shown in Figure 7. Some tearing and deformation features can be observed, which were formed during the fracture process of the samples.

Before this study, the evolution of the sintering performance of Cu nanoparticles with storage time was rarely reported. In previous studies of the preparation of copper nanoparticles, organic additives were widely used as protective or dispersant agents, which could keep the copper particles dispersed or protect them from being oxidized. For example, Dang et al. [16] added PVP as a dispersant and protective agent in a copper sulfate–sodium borohydride system to prepare Cu nanoparticles with a size of 10 nm. The as-prepared Cu nanoparticles could be stored for one week without being oxidized. Balela et al. [34] added gelatin to a copper oxide–hydraulic system as a dispersant and protective agent. The as-prepared Cu nanoparticles, with a size of 50–200 nm were stored in water for 4 months and showed no oxidation peaks. Kanninen et al. [18] added tetraoctylammonium bromide to copper chloride–sodium borohydride. The as-prepared Cu nanoparticles could be stored in sulfolol and an oil–acid solution for four weeks without being oxidized. Although these studies support a positive effect of the organic additives on the oxidation resistance of Cu nanoparticles through XRD analysis, the exact sintering performance of the Cu nanoparticles was not provided. In previous studies of the sintering performance of copper nanoparticles, only the sintering performance of the newly prepared copper particles was reported, as summarized in Table 2. Therefore, based on the current literature, the effect of organic protective agents on the sintering performance of copper particles after storage is still poorly known and needs to be verified.

In this study, we found that the Cu nanoparticles covered with benzimidazole showed relatively good antioxidant properties and could maintain their sintering performance for at least one month. Compared with other organic additives with large molecular weights, such as PVP, the imidazole compounds used in this study were much smaller and therefore could decompose at a lower temperature, which is beneficial for reducing the residual and improving the sintering properties. Some previous studies of organic solderability preservatives [15] indicated that the N atoms of the imidazole compounds could combine with Cu and form complexes, which could prevent the copper from being oxidized.

Compared with the previous coating treatments of Cu nanoparticles, the benzimidazole-coated Cu nanoparticles of this study have the advantages of processing simplicity, low cost, a relatively long preservation time in the air (30 days), and good sintering properties. The method provides an effective approach for the antioxidation of Cu nanoparticles in the microelectronic packaging industry.

## 4. Conclusions

Imidazole-compound-coated Cu nanoparticles with a size of 100–500 nm were prepared via a liquid-phase redox method [44,45] with the addition of imidazole, 2-methylimidazole, 2-phenylimidazole, and benzimidazole protective agents. By complexing with the Cu atoms, the imidazole compounds formed a protective layer on the Cu nanoparticles and thereby improved their oxidation resistance and sintering properties. The Cu nanoparticles prepared with benzimidazole as a protective agent had the best shear strength (32 MPa) after sintering at 300 °C and 2 MPa and could be stored in the air for 30 days without oxidation. These properties meet the basic requirements of the fields of microelectronic packaging and interconnects.

## Figures and Tables

**Table 1:** The measured resistivities of the sintered Cu nanoparticles with different protective agents.

Protective Agent	Resistivity (μΩ·cm)
imidazole	14.7
2-methylimidazole	10.63
2-phenylimidazole	5.69
benzimidazole	4.86
PVP	15.65
without protective agent	30.15

**Table 2:** Summary of the sintering performance of Cu nanoparticles prepared with organic additives.

Diameter (nm)	Organic Additive	Sinter Condition (Atmosphere, Pressure, Temperature)	Shear Strength (MPa)	Ref.
20–110	PVP	air, 5 MPa, 220 °C	13.5	[35]
40–80	PVP	Ar-H2, 1.08 MPa, 300 °C	31.88	[36]
30–270	alkylamine	air, 0 MPa, 350 °C	26	[6]
30	PVP	mixed Ar-H2, 10 MPa, 320 °C	31.2	[5]
50–200	PVP	air, 5 MPa, 250 °C	20	[37]
100	octylamine	N2, 0.4 MPa, 300 °C	17.1	[38]
60–160	1-amino-2-propanol	air, 10 MPa, 250 °C	52.01	[39]
30–80	PVP	mixed Ar-H2, 1.12 MPa, 250 °C	25.41	[40]
54–64	CTAB	H2, 1.2 MPa, 400 °C	37.7	[41]
3.5–9.5	isopropanolamine	Ar, 36.2 MPa 250 °C,	36.2	[42]
80–200	PVP	air, 0.4 MPa, 300 °C	20	[43]

## Data Availability

The data provided in this study are available from the corresponding author upon reasonable request.
